# Water intake and recurrent urinary tract infections prevention: economic impact analysis in seven countries

**DOI:** 10.1186/s12913-023-10234-y

**Published:** 2023-11-03

**Authors:** Juliane Zemdegs, Alison Iroz, Mariacristina Vecchio, Stephane Roze, Yair Lotan

**Affiliations:** 1 Danone Global Research and Inovation Center, Gif-Sur-Yvette, France; 2 Vyoo Agency, Lyon, France; 3 Department of Urology, University of Texas Southwestern Medical Center, 5323 Harry Hines Blvd, Dallas, TX 75390 USA

**Keywords:** Urinary tract infection, Prevention, Drinking water, Water intake, Cost-effectiveness, Budget impact

## Abstract

**Background:**

To estimate the economic impact of preventing urinary tract infections (UTI) by increasing water intake among women with recurrent UTI and low fluid intake across seven countries: France, United Kingdom, Spain, United States of America, Mexico, China and Australia.

**Methods:**

A Markov model was developed to compare costs and outcomes of UTIs associated with low fluid intake in women versus a strategy of primary prevention by increasing water intake. Model inputs were based on randomized controlled trial data which found that increasing water intake by 1.5 L/day decreased the risk of developing cystitis by 48% in women with low fluid intake and recurrent UTI. A time horizon of 10 years was used; outcomes were from the payer perspective and included both direct and indirect costs, reported in 2019 United States dollars ($). Discounting rates varied by country. Scenarios of increasing levels of compliance to the increased water intake strategy were evaluated.

**Results:**

The total cost of one UTI episode, including diagnostics, treatment and complications, ranged from $2164 (Mexico) to $7671 (Australia). Assuming 80% compliance with the increased water intake strategy over a 10-year time horizon, the number of UTIs prevented ranged from 435,845 (Australia) to 24150,272 (China), resulting in total savings of 286 million (Australia) to $4.4 billion (China). Across all countries, increased water intake resulted in lower cost and fewer UTIs compared with low water intake.

**Conclusion:**

Preventing recurrent UTIs by increasing water intake would reduce both the clinical and economic burden associated with UTI. Public, healthcare professionals and patients should be made aware about the preventive positive impact of appropriate water intake on UTIs.

**Supplementary Information:**

The online version contains supplementary material available at 10.1186/s12913-023-10234-y.

## Background

Urinary tract infections (UTI) are common bacterial infections and have an estimated global incidence of 250 million cases/year [1]. Symptoms of UTI can range from mild irritation during urination, to severe systemic illness associated with pyelonephritis, or even death [2]. While both men and women of all ages are at risk of UTIs, they are highly prevalent in women. It is estimated among women with an initial UTI, 20–30% will have a recurrent UTI within 6 months [3]. Women with UTIs also experience decreased quality of life due to pain and general discomfort [4].

In addition to clinical burden, UTIs represent a significant economic burden. The costs attributable to UTIs include both direct costs, such as outpatient doctor visits, diagnostics, antibiotic prescriptions, hospitalization expenses, and indirect costs such as sick days, lost work productivity [2]. A study across five countries in Europe found a mean of 2.78 doctor visits per year, resulting in 3.09 days sick leave due to UTIs in women [4]. Notably, given that UTIs affect a large proportion of women during their peak employment and/or parenting years, the full economic impact of UTI has likely been underestimated to date [5].

Antimicrobial therapy is the current standard of management to prevent recurrence of UTIs, however the specific strategy depends on the number of recurrences experienced and risk factors [6]. In addition to the cost associated with antimicrobial therapy, the degree to which antimicrobials are used for the treatment of UTIs is a contributor to the currently observed increase in antimicrobial resistance [7]. Antimicrobial resistance leads to escalating costs in patient care and increased hospital stays, in addition to an increased risk in mortality [8]. Hence the importance of non-antimicrobial therapy prevention strategies, which may reduce the magnitude of the disease impact on both economy and quality of life. An important modifiable determinant of UTI recurrence is water intake, as increased hydration may be beneficial in the dilution and flushing of bacteriuria [9]. A 12-month open-label randomized controlled trial found that increasing habitual fluid intake by consuming an additional 1.5 L water per day was effective in reducing the risk of recurrence of a UTI by 48% among women with a low baseline fluid intake [10]. Given the feasibility of implementing a simple strategy of increasing water intake, the objective of this study was to assess the economic impact of preventing UTIs by increasing water intake among women with recurrent UTIs and low fluid intake.This analysis was conducted across seven countries, including high-income countries (France, UK, Spain, US, and Australia) and upper-middle-income countries (Mexico and China), to evaluate the potential impact of water intake on UTI prevention across a diverse set of healthcare systems and economic contexts.

By assessing the economic impact of increased water intake as a UTI prevention strategy in a diverse set of countries, this study aims to inform policymakers and healthcare professionals about the potential benefits of promoting this simple, non-pharmacological intervention.

## Methods

A decision analytic Markov model was developed in TreeAge to estimate the cost-effectiveness and budget impact of increased water intake (1.5 L per day over baseline) versus low fluid intake (< 1.5 L per day) on the risk of recurrent UTIs in women. The analysis was performed in a cohort of women with low fluid intake (self-reported as less than 1.5 L of fluid per day) and recurrent UTIs (defined as at least 3 symptomatic UTIs in the past year resulting in a visit to a clinician), from the perspective of the health care payer in seven countries. A time horizon of 10 years was used to capture the impact of increased water intake on long-term morbidity and mortality resulting from UTIs. One-week cycles were used, considering the duration of treatment for an uncomplicated UTI. Costs and effects were discounted based on the recommendations of each country; a summary of the assumptions is provided in TableS1.

### Model design

**Fig. 1:**
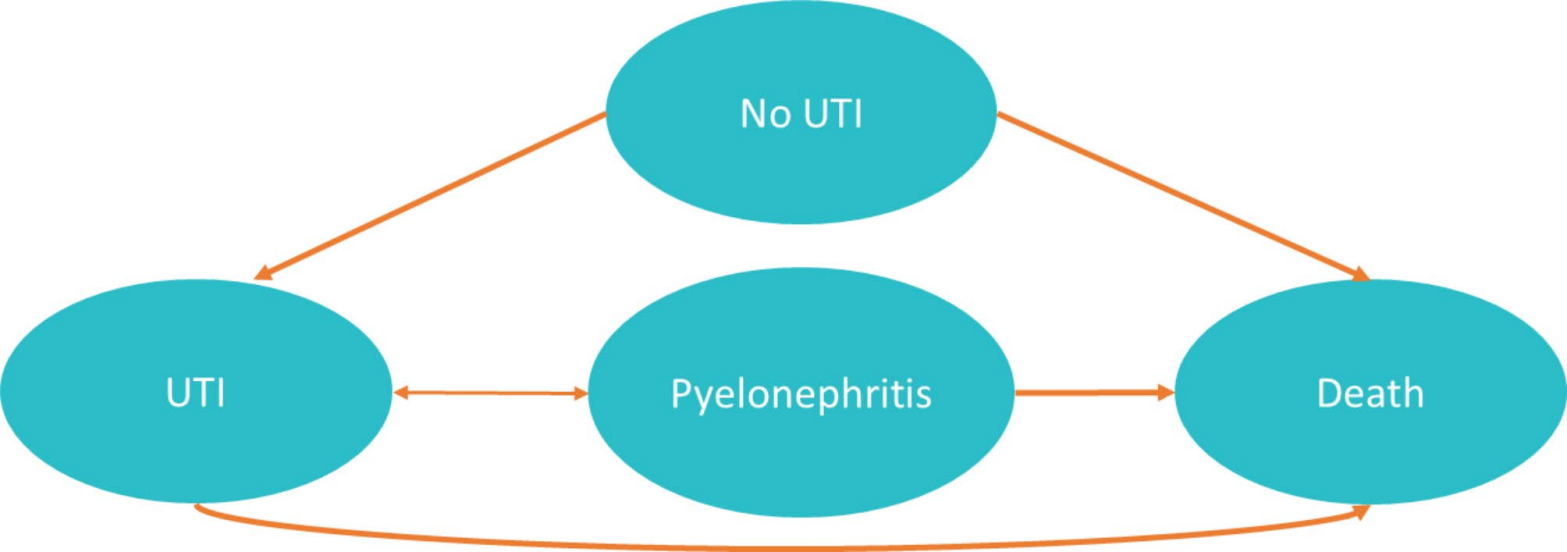
Markov model decision tree of clinical outcomes among patients with recurrent UTI and either a low or high-water intake

The model included four health states: no UTI, lower UTI, upper UTI (pyelonephritis) and death (Fig. 1). All patients enter the “no UTI” state, which assumes that patients in this state have not had a UTI since model start. Patients enter the “lower UTI” health state as soon as they develop one episode of cystitis; if they have no complications or death, they remain in this health state. Patients who have pyelonephritis complication following cystitis transition to “upper UTI” health state. Death is considered an absorbing health state.

### Target population

The target population was women, aged 15 to 65 years old, with low fluid intake and recurrent UTI. The base case assumes a prevalence of 2% for recurrent UTIs [3] and that 35% rate of women have a low fluid intake [11]. The population flow of the model, including the growth per year per country (2018 population), is highlighted in Table S2.

### Costs

Costs were reported in 2019 US dollars (USD); inputs were adjusted for inflation and updated to 2019 using the country-specific consumer price index. Costs were converted in USD assuming an exchange rate as of June 2019 [12]. As stated above, both direct and indirect costs were included, in accordance with country-specific recommendations for disease management and included costs for diagnosis, treatment, complications and productivity loss (Table 1). Antibiotic treatments were costed based on the first recommended treatment; if several first-line treatments were recommended, treatment costs were averaged. Productivity loss was 0.63 days for each UTI [10] and 10.5 days for pyelonephritis [13]. Data on average wages were obtained from the Organization for Economic Co-operation and Development [14], with the exception of China [15] and were used to calculated the productivity losses presented in Table 1.

**Table 1:** Direct and indirect costs per UTI episode, by country (USD)

		France	UK	Spain	US	Mexico	China	Australia
**Diagnosis**	General practitioner	28.06 [28]	60.99 [29]	45.70 [30]	65.43 [31]	41.09 [32]	6.48 [33]	17.20 [34]
	Dipstick	0.77 [28]	0.53 [29]	0.44 [35]	^a^	^a^	^b^	49.94 [34]
	Urinalysis	20.76 [36]	9.28 [29]	4.61 [37]	18.75 [31]	9.63 [32]	^b^	^c^
**Treatment**	Antibiotic	9.89 [36]	8.13 [29]	2.70 [38]	8.25 [39]	19.79 [32]	1.56 [33]	24.22 [34]
**Complication**	Pyelonephritis	3701.95 [3]	5061.86 [29]	3413.78 [35]	2956.40 [31]	2093.46 [32]	2457.71 [40]	7580.00 [34]
**Productivity loss**	UTI	76.95	78.96	67.68	109.18	28.78	18.75	87.45
	Pyelonephritis	1282.47	1315.97	1128.02	1819.75	479.67	312.44	1457.48

UK, United Kingdom; US, United States; USD, United States dollars; UTI, urinary tract infection

^a^Dipstick is not performed in US or Mexico for diagnosis of UTI

^b^The cost of diagnosis is included in the cost of consultation

^c^Urinalysis is not performed in Australia for diagnosis of UTI

^d^Productivity loss calculated assuming 0.63 days and 10.5 days lost for each UTI and pyelonephritis, using the average wages per country

The intervention consisted of increased water intake. For each country, the proportion of women drinking tap water was considered, in addition to the cost per cubic meter of tap and bottled water. The cost of water intake is detailed in Table S3.

### Health-related quality of life

Baseline utility data for France, UK, Spain, US, Mexico and China, was based on Szende et al. [16]; for Australia, baseline utility data was based on Norman et al. [17]. Treatment and management of a UTI was associated with a utility decrement and was applied on a weekly basis as 0.0019 per week for France, UK, Spain, US, Mexico and China [18–20] and 0.0023 per week for Australia [21, 22]. For all countries, the utility decrement associated with pyelonephritis was 0.371 per year [23], applied on the weekly cycle as 0.0071.

### Clinical events

Based on the results of the clinical trial, the mean number of UTI events among women with low fluid intake was 3.2 and among women with increased water intake was 1.7 [10]. Rates were converted into weekly probabilities and were 5.8% per week and 2.8% per week, among low and increased fluid groups, respectively. The risk of developing pyelonephritis was applied equally to both groups for each UTI event at 6% per week [3]. Increased mortality risk due to UTIs was not considered given that the expected impact would be minimal resulting in few patients dying over the time horizon of the model. Transitioning to the death state was based only on general population mortality risk for women only, by country, and was age adjusted [24].

### Analyses

An internal validation was conducted to verify that the model simulated a number of UTIs per patient per year consistent with the randomized controlled trial [10]. Base case analyses considered the direct costs approach including all payers, i.e. health insurance, supplemental health insurance and out of pocket expenses for the patient; indirect costs due to loss of productivity, in addition to direct costs, were considered in a scenario analysis. The cost savings of managing each UTI event with the increased water intake scenario, along with the number of UTI and pyelonephritis events prevent, are presented per country. As compliance to increased water intake strategy was assumed to occur over time, a linear evolution between year 1 (10.0% compliance) and year 10 (80.0% compliance) was applied in the model. Results are presented at difference time horizons, reflecting the varying compliance over time. Both deterministic and probabilistic sensitivity analyses were conducted. In the deterministic analyses, for each country, each variable listed in Table S5 was varied one at a time; in the probabilistic analyses, for each country, all variables listed in Table S6 were varied simultaneously, over 1,000 iterations.

## Results

The number of UTIs and pyelonephritis events prevented, along with the cumulative direct and indirect savings, due to increased fluid intake is presented in Table 2. The number of UTI and pyelonephritis events prevented increased with increasing compliance over the time horizon. The greatest number of events prevented was observed in China. This increase in number of events prevented resulted in an increase in direct and indirect savings across all countries. Cumulative total savings ranged from 286 million USD (Australia) to over 4,4 billion USD (China) at 10 years, with 80% compliance. Across all countries, the number of events prevented and cost savings, were observed even after 1 year, with 10% compliance.

**Table 2:** Cumulative budget savings and number of events prevented with increased water intake strategy (USD)

Country	Time horizon	Compliance	Events prevented (n)		Cumulative savings (USD)		
			UTI	Pyelonephritis	Direct	Indirect	Total
**France**	10 years	80%	1 108 843	61 131	216 528 246,81	156 796 278,79	373 324 525,60
	6 years	50%	397 878	23 873	84 558 500,19	61 232 002,41	145 790 502,60
	1 year	10%	22 374	1 342	4 755 045,31	3 443 307,84	8 198 353,15
**UK**	10 years	80%	1 069 025	64 142	333 903 432,18	168 815 965,11	502 719 397,29
	6 years	50%	413 056	24 783	129 015 577,61	65 228 108,34	194 243 685,95
	1 year	10%	22 926	1 376	7 160 855,44	3 620 408,20	10 781 263,64
**Spain**	10 years	80%	757 688	45 461	171 867 752,89	102 561 980,23	274 429 733,12
	6 years	50%	295 106	17 706	66 939 509,28	39 946 112,71	106 885 621,99
	1 year	10%	16 541	992	3 751 981,02	2 238 992,46	5 990 973,48
**US**	10 years	80%	5 381 380	322 910	850 596 163,56	1 175 235 501,81	2 025 831 665,37
	6 years	50%	2 079 467	124 768	328 658 362,80	454 094 425,15	782 752 787,95
	1 year	10%	115 418	6 925	18 241 789,63	25 203 968,35	43 445 757,98
**Mexico**	10 years	80%	2 204 458	132 267	235 881 902,30	126 881 550,16	362 763 452,46
	6 years	50%	840 495	50 430	89 934 802,16	48 376 187,40	138 310 989,56
	1 year	10%	45 896	2 754	4 910 962,82	2 641 620,95	7 552 583,77
**China**	10 years	80%	24 150 272	1 449 016	3 538 632 908,03	905 577 245,93	4 444 210 153,96
	6 years	50%	9 356 199	561 372	1 370 922 520,26	350 834 989,82	1 721 757 510,08
	1 year	10%	521 003	31 260	76 340 271,64	19 536 361,85	95 876 633,49
**Australia**	10 years	80%	435 845	26 151	209 985 447,23	76 229 554,00	286 215 001,23
	6 years	50%	163 970	9 838	78 999 134,97	28 678 505,60	107 677 640,57
	1 year	10%	8 809	529	4 244 122,04	1 540 714,05	5 784 836,09

UK, United Kingdom; US, United States; USD, United States dollars; UTI, urinary tract infection

The results of the cost-effectiveness analysis for the base case analysis (direct costs only) are presented in Table 3. Across all countries, the increased water intake strategy cost less, resulting in incremental cost savings ranging from $1,065 (Mexico) to $5,850 (Australia). The increased water strategy also resulted in more quality-adjusted life years (0.03 to 0.04). Similar results were observed for the scenario analysis, where both direct and indirect costs are considered (Table S4). Thus, the increased water intake strategy dominates (costs less and is more effective) than usual water intake and can be considered cost-effective.

**Table 3:** Base case cost-effectiveness analyses (USD), by country, over 10-year time horizon

	Usual water intake		Increased water intake		Incremental		ICER
	Costs ($)	QALYs	Costs ($)	QALYs	Costs ($)	QALYs	
France	7,445	7.67	4,571	7.70	-2,873	0.04	Intervention dominates^a^
UK	9,695	7.47	5,623	7.51	-4,071	0.04	Intervention dominates^a^
Spain	6,695	7.92	3,685	7.95	-3,010	0.04	Intervention dominates^a^
US	6,937	7.08	4,169	7.11	-2,767	0.04	Intervention dominates^a^
Mexico	4,645	7.23	3,611	7.27	-1,065	0.03	Intervention dominates^a^
China	3,686	7.56	1,908	7.59	-1,777	0.03	Intervention dominates^a^
Australia	12,953	6.00	7,102	6.03	-5,850	0.03	Intervention dominates^a^

^a^ Increased water intake costs less and is more effective than usual water intake

ICER, incremental cost-effectiveness ratio; QALY, quality-adjusted life year; UK, United Kingdom; US, United States; USD, United States dollars

Deterministic sensitivity analyses, along with the results, are presented in Table S5. Across the parameters evaluated, increased water intake cost less and was more effective for both the lower and upper bound estimates. Probabilistic sensitivity analyses found that across all iterations for the US, Spain, and France, increased water intake cost less and was more effective (Figures S1-S3). For the UK, Mexico, China and Australia, while increased water intake was also more effective, in less than 5% of iterations, it cost more (Figures S4 – S7).

## Discussion

Using a decision analytic model, we evaluated the cost-effectiveness and budget impact of increased water intake in the prevention of recurrent UTIs in women with low fluid intake and a history of recurrent UTIs. The model revealed that compared to lower water intake, increasing water had a significant economic impact across all seven countries by decreasing the costs associated with UTI events. Further, considerable cumulative total cost savings were observed when a greater proportion of the population complied with the increased water intake recommendation.

There is widespread concern about growing healthcare costs and evaluating the economic impact of prevention strategies has gained importance. To consider whether a public health strategy is worthwhile, specific factors are considered, including disease prevalence, morbidity and costs, as well as the cost-effectiveness and the budget impact of the health strategy. In addition to the high clinical burden placed on women with recurrent UTI, our study found that recurrent UTIs represents a significant societal burden, with estimated indirect costs ranging from 59 million (Australia) to 911 million (US), further highlighting the importance of UTI disease prevention.

To the best of our knowledge, only one study has previously evaluated the economic impact of preventing UTIs using an increased water intake strategy. In 2015, Bruyère et al. [3] reported that if 100% of the general French population increased their water intake, 2.77 million UTI episodes would be prevented with an annual cost-saving of €2.2 billion. While both studies demonstrate the cost savings associated with increased water intake, the results reported here report lower cost savings, which may be due to differences in model assumptions between the two studies. Bruyère et al. [3] derived the risk reduction in UTIs via increased water intake from a combination of one observational study and a small interventional trial, among both men and women, resulting in high recurrent rates of UTIs (5.3 − 30%) and a reduction in the risk of developing recurrent UTIs of 33% with increased water. In our study, UTI recurrent risk reduction was lower, however, the data was based on a large 12-month randomized controlled trial including 140 women [10], providing a more robust estimation of the benefit of increasing water intake on UTI prevention.

The clinical and budget impact of our results also rely on global evidence that a substantial proportion of women do not drink enough water, as compared to public health authorities’ recommendations. In fact, studies have shown that 35% of adult women drink less than 1,5 L/day of fluids, while 40% of women drink less than 0,5 L/day of water [11, 25]. However, even under poor compliance assumptions of 10%, our model still estimated total cost savings. As observed in this study, compliance with increasing water intake has an important impact on cost-effectiveness with high compliance levels substantially increasing the number of UTI prevented. In order to achieve higher compliance rates, public health interventions, such as awareness campaigns and health education programs should be considered, communicating the importance of appropriate water intake.

The impact of our results should also be interpreted in the context of the well documented increasing rates of antibiotic resistance in patients with recurrent UTIs [26]. Substantial variations exist in antibiotic prescription practices in different countries, and yet, some clinicians and patients might not perceive antibiotic resistance as a reason to refrain from antibiotic use [27]. In this context, the World Health Organization organizes a yearly campaign to increase awareness of antimicrobial resistance and encourages best practices and responsible use among the general public, health care practitioners and policy makers through effective communication, education and training.

Our study has multiple strengths. First, our analysis considered multiple outcomes (cost-effectiveness and budget impact) across multiple countries, demonstrating the value of increased water intake across different healthcare systems with varying costs. Second, our study population included only those who would benefit from the increasing water intake strategy, by targeting women with recurrent UTI and low fluid intake. We also considered that behavior change takes time, thus, we evaluated different proportions of the population adhering to the increased water intake strategy through compliances of 10%, 50% and 80%. Finally, our study may be considered conservative as the larger impact of reduced health care resources in the context of the health system were not considered; future research may wish to quantify this impact. Our study also has limitations. As noted above, risk reduction values were based on a single long-term randomized controlled trial investigating the effects of increasing water intake on UTI risk; to the best of our knowledge, this is the only available long-term evidence available. Further research should be performed to assess impact of increased water intake in other settings with variable geography and economic conditions. The countries we selected were high-income and upper-middle-income countries, which may limit generalizability to low-income countries. As UTI-specific quality of life measures were not available, we used generic health-related quality of life measures; it is unlikely that these utility measures would greatly differ, thus resulting in minimal impact on the results. Further, costs related to the implementation of public health awareness and prevention programs were not considered in our analysis; the cost of such programs or initiatives should be assessed in the future. There is also a possibility that the economic impact of increased water intake was underestimated in the current manuscript. First, to calculate our target population we used a conservative estimation of recurrent UTI prevalence based on the French population as prevalence of recurrent UTI in regions other than the USA and France are not available in the scientific literature. Secondly, to calculate the total number of UTIs per country we used the number of recurrent UTI episodes experienced in the general female population. As our target population included only women who are low drinkers, it is likely that these women experience a higher-than-average number of recurrent UTI episodes as compared to the general female population. Taking into consideration our conservative calculation for both the target population and number of UTI episodes per country, increased water intake may have a greater economic impact then as reported here.

## Conclusions

Our cost-effectiveness and budget impact analysis provides meaningful data on the significant cost savings of preventing recurrent UTI through increased water intake across seven countries. As compliance to increased water intake is crucial to attain the clinical benefit and cost savings observed herein, public awareness emphasizing the impact of increased water intake is encouraged.

### Electronic supplementary material

Below is the link to the electronic supplementary material.

Supplementary Material 1

## Data Availability

All data generated or analyzed during this study are included in this published article and its supplementary information files.

## List of abbreviations

UK: United Kingdom
US: United States
USD: United States dollars
UTI: Urinary tract infection
