# Supplementary material for: Water intake and recurrent urinary tract infections prevention: economic impact analysis in seven countries
Source: BMC Health Serv Res. 2023 Nov 3;23:1197. doi: 10.1186/s12913-023-10234-y (PMC10623695; doi:10.1186/s12913-023-10234-y)
Supplement: Supplementary file 1 — Supplementary Material 1 [file 12913_2023_10234_MOESM1_ESM.docx]

**Table S1. Summary of assumptions for the CEM**

|  | **Methodological choice** | **Rationale** |
| --- | --- | --- |
| **Perspective** | Direct costs (all payers) | This perspective includes health insurance, supplemental health insurance and out of pocket expenses for the patient. Direct and indirect costs are presented as a scenario analysis. |
| **Time horizon** | 10 years | Sufficient to capture all costs and effects of intervention |
| **Discount rate** | France: 2.5% (1)  UK: 3.5% (2)  Spain: 3% (3)  USA: 3% (3)  Mexico: 5% (4)  China: 5% (5)  Australia: 5% (3) | Recommendations of each country |
| **Comparator** | Low water intake versus increased water intake | Based on clinical trial |
| **Costs** | Screening costs, antibiotic treatment costs, complication costs, cost of water in line with each country | Reflects UTI management |
| **Outcome** | Incremental cost effectiveness ratio | Reflects standard approach |

CEM, cost-effectiveness model; UK, United Kingdom; USA, United States of America; UTI, urinary tract infection

**Table S2. Population flow by country (2018 population)**

| **Country** | **Total population** | **Women, aged 15-65 years** | **Recurrent UTI prevalence (2%)** | **Prevalence low drinkers (35%)** | **Population growth per year** |
| --- | --- | --- | --- | --- | --- |
| **France** | 66,987,244 | 20,642,919 | 412,858 | 144,500 | 0.2% |
| **UK** | 66,488,991 | 21,152,095 | 423,042 | 148,065 | 0.6% |
| **Spain** | 46,723,749 | 15,260,792 | 305,216 | 106,826 | 0.3% |
| **USA** | 327,167,434 | 106,487,206 | 2,129,744 | 745,410 | 0.6% |
| **Mexico** | 126,190,788 | 42,344,451 | 846,889 | 296,411 | 1.1% |
| **Australia** | 24,992,369 | 8,127,406 | 162,548 | 56,892 | 1.6% |
| **China** | 1,393,000,000 | 480,687,262 | 9,613,745 | 3,364,811 | 0.5% |

UK, United Kingdom; USA, United States; UTI, urinary tract infection

**Table S3. Cost of water intake per country (USD)**

| **Country** | **Proportion drinking tap water** | **Cost per cubic meter of tap water** | **Cost per cubic meter of bottled water(6)** | **Water cost per cubic meter used in model** |
| --- | --- | --- | --- | --- |
| France | 41%(7) | $2.34(8) | $180.80 | $106.95 |
| UK | 72%(7) | $1.81(8) | $378.03 | $108.85 |
| Spain | 66%(7) | $1.10(8) | $142.45 | $48.69 |
| Mexico | 2%(7) | $0.58(9) | $175.32 | $171.83 |
| Australia | 74%(10) | $0.82(11) | $383.51 | $99.67 |
| China | 91%(7) | $1.59(12) | $142.45 | $13.90 |
| US | 61%(13) | $1.23(8) | $235.59 | $92.89 |

UK, United Kingdom; USA, United States; USD, United States dollars

**Table S4.** Scenario analysis including both direct and indirect costs (USD), by country, over 10-year time horizon.

|  | **Usual water intake** | | **Increased water intake** | | **Incremental** | | **ICER** |
| --- | --- | --- | --- | --- | --- | --- | --- |
|  | **Costs ($)** | **QALYs** | **Costs ($)** | **QALYs** | **Costs ($)** | **QALYs** |  |
| France | 11,513 | 7.67 | 6,555 | 7.70 | -4,959 | 0.04 | Intervention dominates^a^ |
| UK | 13,696 | 7.47 | 7,574 | 7.51 | -6,122 | 0.04 | Intervention dominates^a^ |
| Spain | 10,203 | 7.92 | 5,395 | 7.95 | -4,808 | 0.04 | Intervention dominates^a^ |
| US | 14,117 | 7.08 | 7,670 | 7.11 | -6,447 | 0.04 | Intervention dominates^a^ |
| Mexico | 6,070 | 7.23 | 4,306 | 7.27 | -1,764 | 0.03 | Intervention dominates^a^ |
| China | 4,574 | 7.56 | 2,342 | 7.59 | -2,233 | 0.03 | Intervention dominates^a^ |
| Australia | 17,101 | 6.00 | 9,124 | 6.03 | -7,976 | 0.03 | Intervention dominates^a^ |

^a^ Increased water intake costs less and is more effective than usual water intake

ICER, incremental cost-effectiveness ratio; QALY, quality-adjusted life year; UK, United Kingdom; US, United States; USD, United States dollars.

**Table S5.** Deterministic sensitivity analyses (USD), by country.

|  | **Value in**  **base case** | **Value tested** | | **ICER** | |
| --- | --- | --- | --- | --- | --- |
|  |  | **Lower bound** | **Upper bound** | **Lower bound** | **Upper bound** |
| **France** |  |  |  |  |  |
| Time horizon | 10 years | 3 years | 5 years | Intervention dominates^a^ | Intervention dominates^a^ |
| Start age | 35.7 years | 28.6 years | 42.8 years | Intervention dominates^a^ | Intervention dominates^a^ |
| Discount rate | 2.5% | 0% | 4% | Intervention dominates^a^ | Intervention dominates^a^ |
| Number of UTIs for usual water intake | 3.2 | -20% | +20% | Intervention dominates^a^ | Intervention dominates^a^ |
| Relative risk of UTI increased versus usual water intake | 0.48 | -20% | +20% | Intervention dominates^a^ | Intervention dominates^a^ |
| Risk of pyelonephritis | 6% | -20% | +20% | Intervention dominates^a^ | Intervention dominates^a^ |
| Water cost | Table S3 | -20% | +20% | Intervention dominates^a^ | Intervention dominates^a^ |
| Percentage of tap water | Table S3 | -20% | +20% | Intervention dominates^a^ | Intervention dominates^a^ |
| **UK** |  |  |  |  |  |
| Time horizon | 10 years | 3 years | 5 years | Intervention dominates^a^ | Intervention dominates^a^ |
| Start age | 35.7 years | 28.6 years | 42.8 years | Intervention dominates^a^ | Intervention dominates^a^ |
| Discount rate | 3.5% | 0% | 5% | Intervention dominates^a^ | Intervention dominates^a^ |
| Number of UTIs for usual water intake | 3.2 | -20% | +20% | Intervention dominates^a^ | Intervention dominates^a^ |
| Relative risk of UTI increased versus usual water intake | 0.48 | -20% | +20% | Intervention dominates^a^ | Intervention dominates^a^ |
| Risk of pyelonephritis | 6% | -20% | +20% | Intervention dominates^a^ | Intervention dominates^a^ |
| Water cost | Table S3 | -20% | +20% | Intervention dominates^a^ | Intervention dominates^a^ |
| Percentage of tap water | Table S3 | -20% | +20% | Intervention dominates^a^ | Intervention dominates^a^ |
| **Spain** |  |  |  |  |  |
| Time horizon | 10 years | 3 years | 5 years | Intervention dominates^a^ | Intervention dominates^a^ |
| Start age | 35.7 years | 28.6 years | 42.8 years | Intervention dominates^a^ | Intervention dominates^a^ |
| Discount rate | 3% | 0% | 5% | Intervention dominates^a^ | Intervention dominates^a^ |
| Number of UTIs for usual water intake | 3.2 | -20% | +20% | Intervention dominates^a^ | Intervention dominates^a^ |
| Relative risk of UTI increased versus usual water intake | 0.48 | -20% | +20% | Intervention dominates^a^ | Intervention dominates^a^ |
| Risk of pyelonephritis | 6% | -20% | +20% | Intervention dominates^a^ | Intervention dominates^a^ |
| Water cost | Table S3 | -20% | +20% | Intervention dominates^a^ | Intervention dominates^a^ |
| Percentage of tap water | Table S3 | -20% | +20% | Intervention dominates^a^ | Intervention dominates^a^ |
| **US** |  |  |  |  |  |
| Time horizon | 10 years | 3 years | 5 years | Intervention dominates^a^ | Intervention dominates^a^ |
| Start age | 35.7 years | 28.6 years | 42.8 years | Intervention dominates^a^ | Intervention dominates^a^ |
| Discount rate | 3% | 0% | 5% | Intervention dominates^a^ | Intervention dominates^a^ |
| Number of UTIs for usual water intake | 3.2 | -20% | +20% | Intervention dominates^a^ | Intervention dominates^a^ |
| Relative risk of UTI increased versus usual water intake | 0.48 | -20% | +20% | Intervention dominates^a^ | Intervention dominates^a^ |
| Risk of pyelonephritis | 6% | -20% | +20% | Intervention dominates^a^ | Intervention dominates^a^ |
| Water cost | Table S3 | -20% | +20% | Intervention dominates^a^ | Intervention dominates^a^ |
| Percentage of tap water | Table S3 | -20% | +20% | Intervention dominates^a^ | Intervention dominates^a^ |
| **Mexico** |  |  |  |  |  |
| Time horizon | 10 years | 3 years | 5 years | Intervention dominates^a^ | Intervention dominates^a^ |
| Start age | 35.7 years | 28.6 years | 42.8 years | Intervention dominates^a^ | Intervention dominates^a^ |
| Discount rate | 5% | 0% | 7% | Intervention dominates^a^ | Intervention dominates^a^ |
| Number of UTIs for usual water intake | 3.2 | -20% | +20% | Intervention dominates^a^ | Intervention dominates^a^ |
| Relative risk of UTI increased versus usual water intake | 0.48 | -20% | +20% | Intervention dominates^a^ | Intervention dominates^a^ |
| Risk of pyelonephritis | 6% | -20% | +20% | Intervention dominates^a^ | Intervention dominates^a^ |
| Water cost | Table S3 | -20% | +20% | Intervention dominates^a^ | Intervention dominates^a^ |
| Percentage of tap water | Table S3 | -20% | +20% | Intervention dominates^a^ | Intervention dominates^a^ |
| **China** |  |  |  |  |  |
| Time horizon | 10 years | 3 years | 5 years | Intervention dominates^a^ | Intervention dominates^a^ |
| Start age | 35.7 years | 28.6 years | 42.8 years | Intervention dominates^a^ | Intervention dominates^a^ |
| Discount rate | 5% | 0% | 8% | Intervention dominates^a^ | Intervention dominates^a^ |
| Number of UTIs for usual water intake | 3.2 | -20% | +20% | Intervention dominates^a^ | Intervention dominates^a^ |
| Relative risk of UTI increased versus usual water intake | 0.48 | -20% | +20% | Intervention dominates^a^ | Intervention dominates^a^ |
| Risk of pyelonephritis | 6% | -20% | +20% | Intervention dominates^a^ | Intervention dominates^a^ |
| Water cost | Table S3 | -20% | +20% | Intervention dominates^a^ | Intervention dominates^a^ |
| Percentage of tap water | Table S3 | -20% | +20% | Intervention dominates^a^ | Intervention dominates^a^ |
| **Australia** |  |  |  |  |  |
| Time horizon | 10 years | 3 years | 5 years | Intervention dominates^a^ | Intervention dominates^a^ |
| Start age | 35.7 years | 28.6 years | 42.8 years | Intervention dominates^a^ | Intervention dominates^a^ |
| Discount rate | 5% | 0% | 3.5% | Intervention dominates^a^ | Intervention dominates^a^ |
| Number of UTIs for usual water intake | 3.2 | -20% | +20% | Intervention dominates^a^ | Intervention dominates^a^ |
| Relative risk of UTI increased versus usual water intake | 0.48 | -20% | +20% | Intervention dominates^a^ | Intervention dominates^a^ |
| Risk of pyelonephritis | 6% | -20% | +20% | Intervention dominates^a^ | Intervention dominates^a^ |
| Water cost | Table S3 | -20% | +20% | Intervention dominates^a^ | Intervention dominates^a^ |
| Percentage of tap water | Table S3 | -20% | +20% | Intervention dominates^a^ | Intervention dominates^a^ |

^a^ Increased water intake costs less and is more effective than usual water intake

ICER, incremental cost-effectiveness ratio; QALY, quality-adjusted life year; UK, United Kingdom; US, United States; USD, United States dollars; UTI, urinary tract infection

**Table S6.** Deterministic sensitivity analyses (USD), by country.

|  | **Value in**  **base case** | **Standard error** | **Distribution** | |
| --- | --- | --- | --- | --- |
| **France** |  |  | |  |
| Start age | 35.7 years | 0.71 | | Normal |
| Number of UTIs for usual water intake | 3.2 | 0.10 | | Normal |
| Number of UTIs for increased water intake | 1.7 | 0.08 | | Normal |
| Risk of pyelonephritis | 6% | 0.24 | | Beta |
| Cost of bottled water per cubic meter | $180.80 | $18.08* | | Gamma |
| Prevalence low water drinker | 35% | 2% | | Beta |
| Percentage drinking bottled water | 59% | 5.9%* | | Beta |
| **UK** |  |  | |  |
| Start age | 35.7 years | 0.71 | | Normal |
| Number of UTIs for usual water intake | 3.2 | 0.10 | | Normal |
| Number of UTIs for increased water intake | 1.7 | 0.08 | | Normal |
| Risk of pyelonephritis | 6% | 0.24 | | Beta |
| Cost of bottled water per cubic meter | $378.03 | $37.80* | | Gamma |
| Prevalence low water drinker | 35% | 2% | | Beta |
| Percentage drinking bottled water | 28% | 2.8* | | Beta |
| **Spain** |  |  | |  |
| Start age | 35.7 years | 0.71 | | Normal |
| Number of UTIs for usual water intake | 3.2 | 0.10 | | Normal |
| Number of UTIs for increased water intake | 1.7 | 0.08 | | Normal |
| Risk of pyelonephritis | 6% | 0.24 | | Beta |
| Cost of bottled water per cubic meter | $142.45 | $14.25* | | Gamma |
| Prevalence low water drinker | 35% | 2% | | Beta |
| Percentage drinking bottled water | 34% | 3.4% | | Beta |
| **US** |  |  | |  |
| Start age | 35.7 years | 0.71 | | Normal |
| Number of UTIs for usual water intake | 3.2 | 0.10 | | Normal |
| Number of UTIs for increased water intake | 1.7 | 0.08 | | Normal |
| Risk of pyelonephritis | 6% | 0.24 | | Beta |
| Cost of bottled water per cubic meter | $235.59 | $23.56* | | Gamma |
| Prevalence low water drinker | 35% | 2% | | Beta |
| Percentage drinking bottled water | 39% | 3.9% | | Beta |
| **Mexico** |  |  | |  |
| Start age | 35.7 years | 0.71 | | Normal |
| Number of UTIs for usual water intake | 3.2 | 0.10 | | Normal |
| Number of UTIs for increased water intake | 1.7 | 0.08 | | Normal |
| Risk of pyelonephritis | 6% | 0.24 | | Beta |
| Cost of bottled water per cubic meter | $175.32 | $17.53* | | Gamma |
| Prevalence low water drinker | 35% | 2% | | Beta |
| Percentage drinking bottled water | 98% | 9.8% | | Beta |
| **China** |  |  | |  |
| Start age | 35.7 years | 0.71 | | Normal |
| Number of UTIs for usual water intake | 3.2 | 0.10 | | Normal |
| Number of UTIs for increased water intake | 1.7 | 0.08 | | Normal |
| Risk of pyelonephritis | 6% | 0.24 | | Beta |
| Cost of bottled water per cubic meter | $142.45 | $14.25* | | Gamma |
| Prevalence low water drinker | 35% | 2% | | Beta |
| Percentage drinking bottled water | 9% | 0.9% | | Beta |
| **Australia** |  |  | |  |
| Start age | 35.7 years | 0.71 | | Normal |
| Number of UTIs for usual water intake | 3.2 | 0.10 | | Normal |
| Number of UTIs for increased water intake | 1.7 | 0.08 | | Normal |
| Risk of pyelonephritis | 6% | 0.24 | | Beta |
| Cost of bottled water per cubic meter | $383.51 | $38.35* | | Gamma |
| Prevalence low water drinker | 35% | 2% | | Beta |
| Percentage drinking bottled water | 26% | 2.6% | | Beta |

*Assumed to be 10% of the mean

UK, United Kingdom; US, United States; USD, United States dollars; UTI, urinary tract infection

**Figure S1. ICER scatterplot for US, increased versus low water intake**


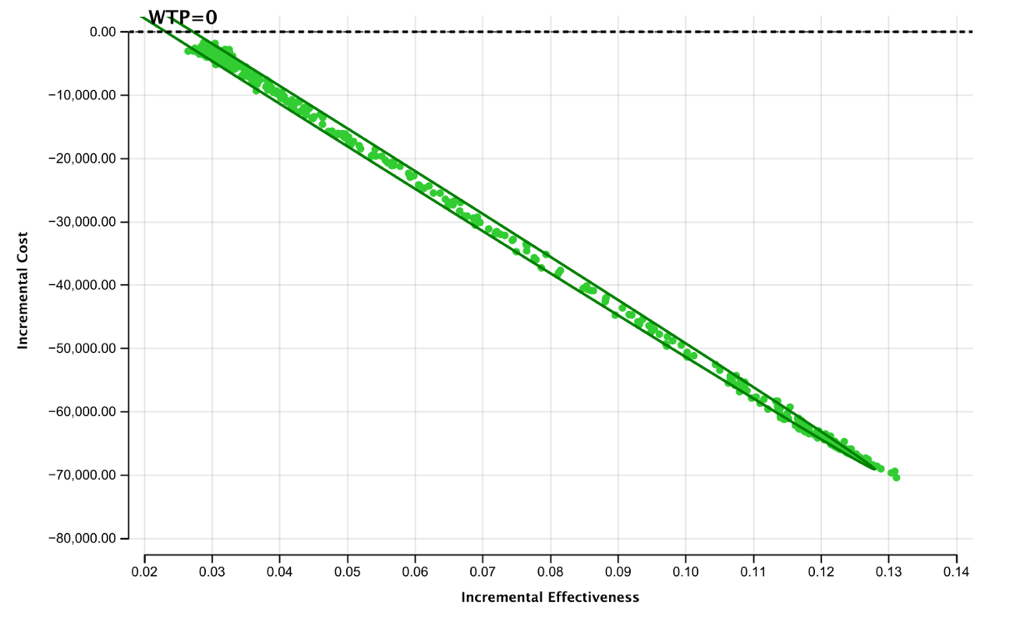


ICER, incremental cost-effectiveness ratio ; US, United States

**Figure S2. ICER scatterplot for Spain, increased versus low water intake**


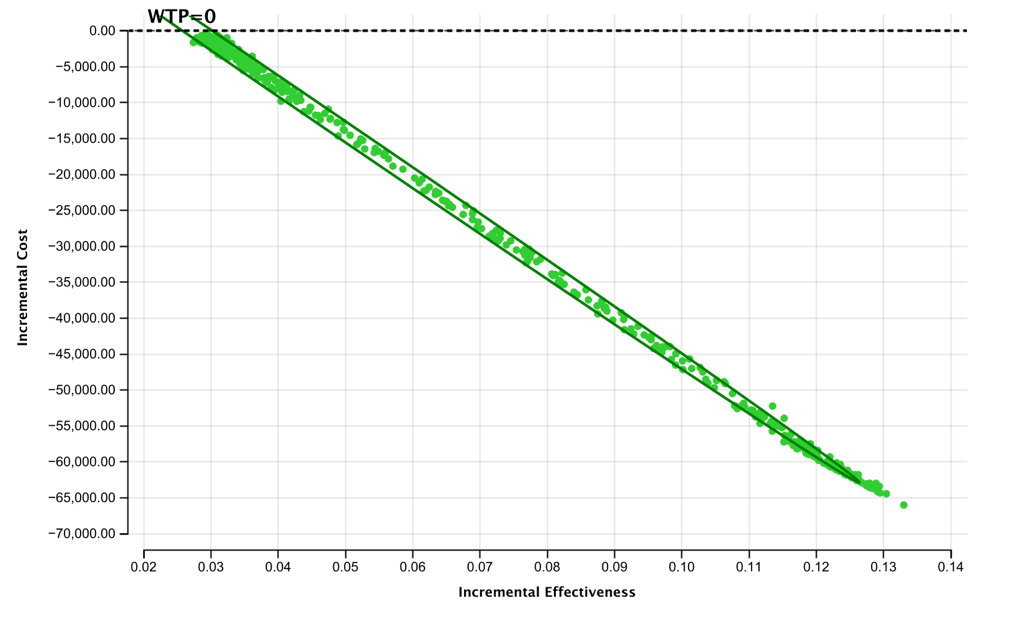


ICER, incremental cost-effectiveness ratio

**Figure S3. ICER scatterplot for France, increased versus low water intake**


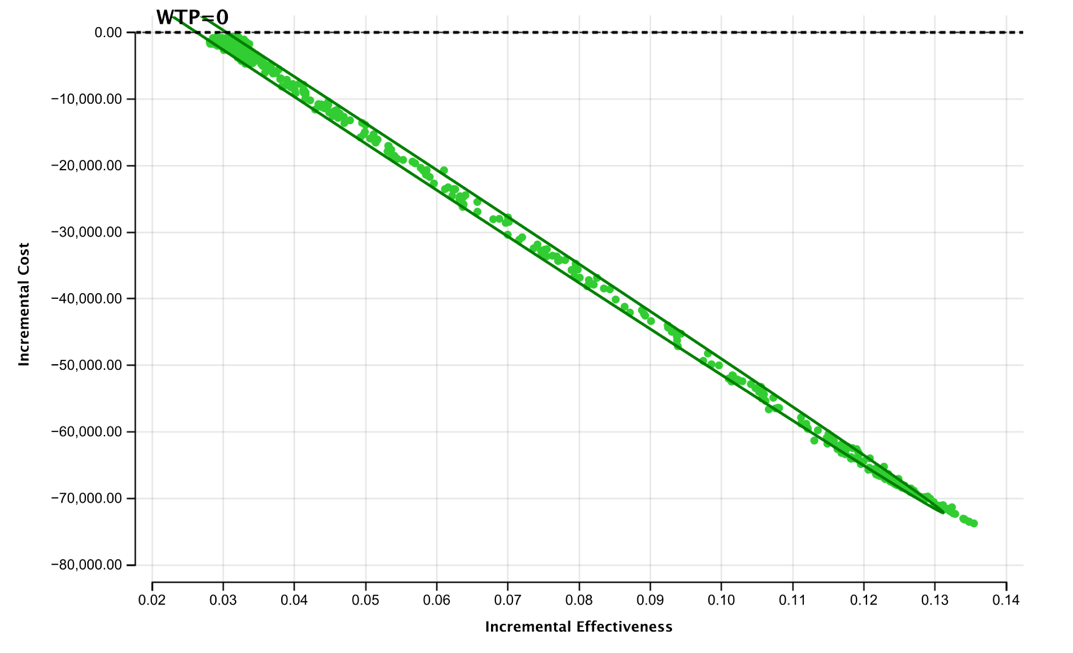


ICER, incremental cost-effectiveness ratio

**Figure S4. ICER scatterplot for UK, increased versus low water intake**


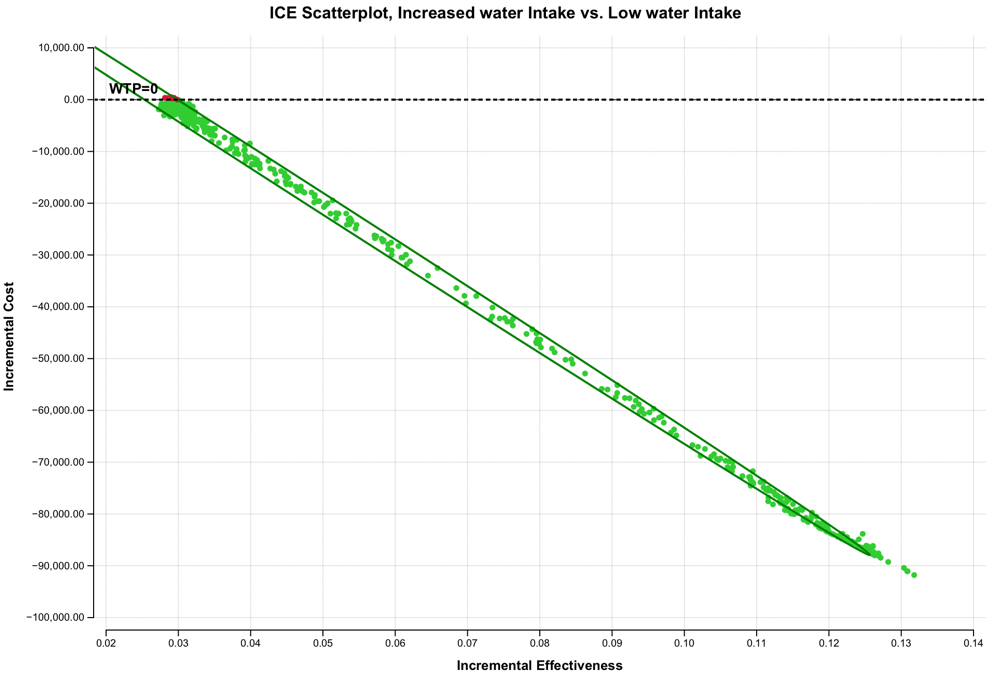


ICER, incremental cost-effectiveness ratio ; UK, United Kingdom

**Figure S5. ICER scatterplot for Mexico, increased versus low water intake**


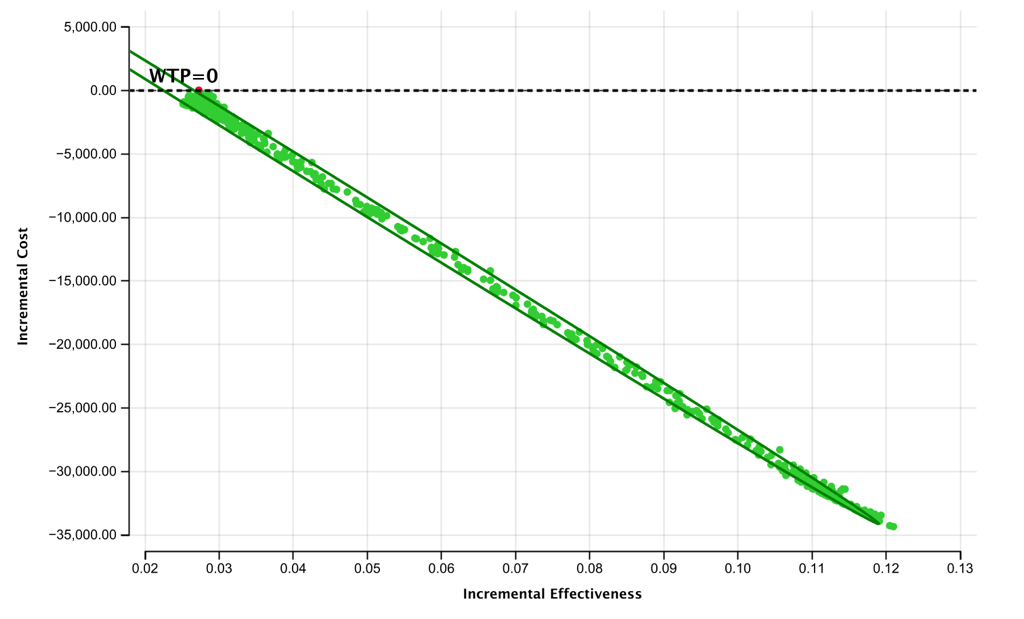


ICER, incremental cost-effectiveness ratio

**Figure S6. ICER scatterplot for China, increased versus low water intake**


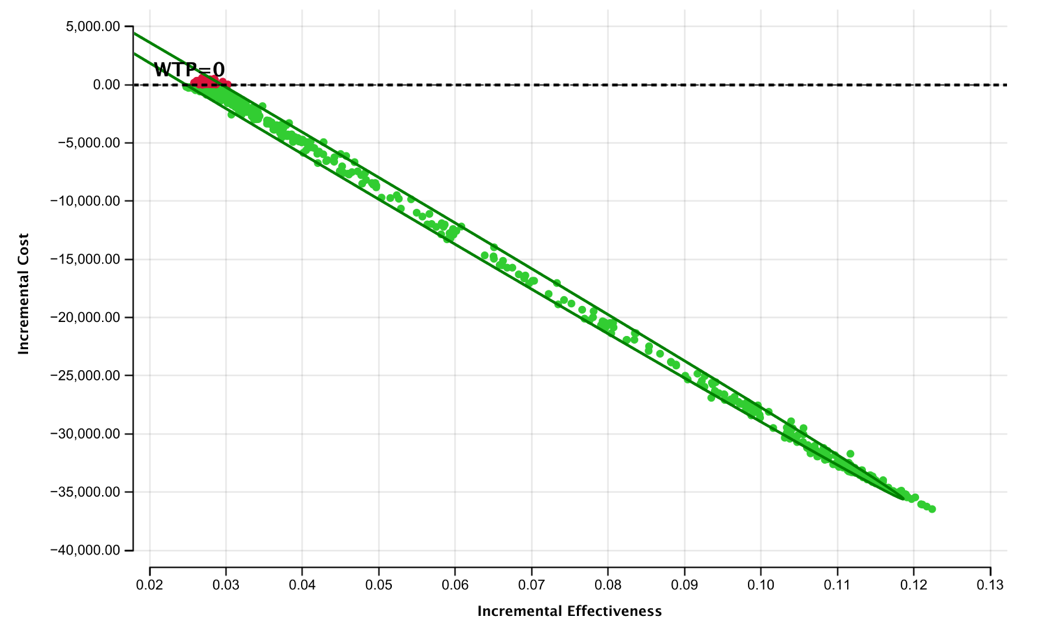


ICER, incremental cost-effectiveness ratio

**Figure S7. ICER scatterplot for Australia, increased versus low water intake**


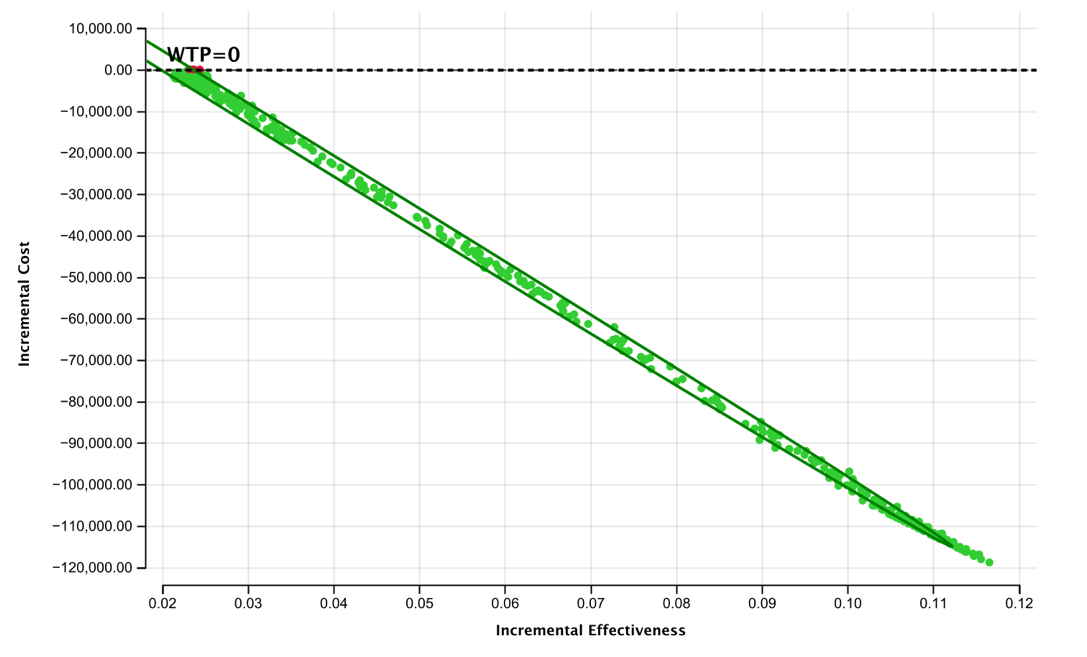


ICER, incremental cost-effectiveness ratio

1. Haute Autorité de Santé. Choices in methods for economic evaluation - HAS. 2020.

2. National Institute for Health and Care Excellence. CHTE methods review: Discounting. Task and finish group report.; 2020.

3. Sharma D, Aggarwal AK, Downey LE, Prinja S. National Healthcare Economic Evaluation Guidelines: A Cross-Country Comparison. Pharmacoecon Open. 2021;5(3):349-64.

4. Daccache C, Rizk R, Dahham J, Evers S, Hiligsmann M, Karam R. Economic evaluation guidelines in low- and middle-income countries: a systematic review. Int J Technol Assess Health Care. 2021;38(1):e1.

5. ISPOR. Pharmacoeconomic Guidelines: China Mainland April 22, 2021 [Available from: <https://www.ispor.org/heor-resources/more-heor-resources/pharmacoeconomic-guidelines/pe-guideline-detail/china-mainland>.

6. Mintel. Market shares and bottled water prices 2012 [Available from: <https://marketsizes.mintel.com/query/201726841/performance/market>.

7. Guelinckx I, Ferreira-Pego C, Moreno LA, Kavouras SA, Gandy J, Martinez H, et al. Intake of water and different beverages in adults across 13 countries. Eur J Nutr. 2015;54 Suppl 2:45-55.

8. OECD. ENVIRONMENT AT A GLANCE 2013: OECD INDICATORS. International Water Association. 2013. <https://www.oecd-ilibrary.org/docserver/9789264185715-10-en.pdf?expires=1570002018&id=id&accname=guest&checksum=28EA75BFACFD512418123CD0506BAD8A> [

9. Comisión Nacional del Agua. Tarifas de agua potable y saneamiento para uso doméstico tipo residencial. 2017. <http://sina.conagua.gob.mx/sina/tema.php?tema=tarifas&ver=reporte> [

10. Australian Bureau of Statistics. 4364.0.55.012 Australian Health Survey: Consumption of Food Groups from the Australian Dietary Guidelines, 2011-12 2011 [Available from: <https://www.abs.gov.au/ausstats/abs@.nsf/Lookup/by%20Subject/4364.0.55.012~2011-12~Main%20Features~Water~10001>.

11. Sydney Water. Water prices for your home. 2019. <http://www.sydneywater.com.au/SW/accounts-billing/understanding-your-bill/prices-for-your-home/index.htm> [

12. The World Bank. World Bank Analytical and Advi sory Assistance (AAA) Program China: Addressing Water Scarcity – From Analysis to Action Policy Note Water supply pricing in China Economic Efficiency, Environment, and social affordability. 2013.

13. Drewnowski A, Rehm CD, Constant F. Water and beverage consumption among adults in the United States: cross-sectional study using data from NHANES 2005-2010. BMC Public Health. 2013;13:1068.
